# Two Fœtuses of Unequal Size at the Same Abortion

**Published:** 1850-11

**Authors:** F. Brady

**Affiliations:** Belle Fountain, Mehaska Co., Iowa


### ARTICLE VI.

*Two Fœtuses of unequal size at the same Abortion.*—By Dr. F. Brady, of Belle Fountain, Mehaska Co., Iowa.

Aug. 13th, 1850—Called in conjunction with E. A. B., M. D., in haste to see a lady, Mrs. S., aged about 22, who had labored under a spinal affection for a number of years. She was represented by her husband to have been delivered of a fœtus some three hours previously; placenta not delivered. When we arrived we found her comparatively easy; no labor pains, the os uteri very much contracted. We tried in vain all the ordinary means, except the ergot, to excite uterine contraction; we then resorted to the secale coruntum; after exhibiting about twice the ordinary quantity pains came on and ultimately dilatation of the os uteri, so as to expel a small mass, which, on examination, proved to be another fœtus, from four to five inches in length, of ordinary proportions, vitality evidently having been destroyed within a few hours, (we omitted to mention that the first fœtus was about 12 inches in length, very much decomposed, with a very nauseous odor, evidently having been dead for some time; I think about a month, as she had a violent uterine hemorrhage about that time, and had been very much indisposed since). After considerable labor we succeeded in removing the small placenta, and small portions of what we supposed to be the larger one. The parts being very sensitive, we deemed it advisable to desist in our efforts to produce expulsion. In about an hour, however, she was delivered of a homogeneous mass apparently of very different texture and color from an ordinary placenta, about four inches in length, two in breadth, one and a half in thickness. It was attended with very little hemorrhage. She has since done well and has pretty nearly recovered. I learned from her husband that this was her third abortion. She never had a living child.

Queries.—Could both of them have been conceived at the same time, one remaining comparatively dormant while the other grew to the specified size? or could they have been conceived at different times? What could have caused the placenta, if such it was, to assume that shape and appearance? These are questions I feel myself inadequate to solve.

